# Initial Feasibility and Acceptability of a Comprehensive Intervention for Methamphetamine-Using Pregnant Women in South Africa

**DOI:** 10.1155/2014/929767

**Published:** 2014-01-05

**Authors:** Hendrée E. Jones, Bronwyn Myers, Kevin E. O'Grady, Stefan Gebhardt, Gerhard B. Theron, Wendee M. Wechsberg

**Affiliations:** ^1^UNC Horizons Program, Department of Obstetrics and Gynecology, School of Medicine, University of North Carolina at Chapel Hill, 400 Roberson Street, Carrboro, NC 27510, USA; ^2^Departments of Psychiatry and Behavioral Sciences and Obstetrics and Gynecology, School of Medicine, Johns Hopkins University, Baltimore, MD 21224, USA; ^3^Alcohol and Drug Abuse Research Unit, Medical Research Council, Tygerberg, Cape Town 7505, South Africa; ^4^Department of Psychiatry and Mental Health, University of Cape Town, Cape Town 7700, South Africa; ^5^Department of Psychology, University of Maryland, College Park, College Park, MD 20742, USA; ^6^Department of Obstetrics and Gynaecology, Stellenbosch University, Stellenbosch 7600, South Africa; ^7^Substance Abuse Treatment Evaluations and Interventions Research Program, RTI International, Research Triangle Park, NC 27709, USA

## Abstract

The purpose of the present study was to determine the feasibility, acceptability, and initial efficacy of a women-focused intervention addressing methamphetamine use and HIV sexual risk among pregnant women in Cape Town, South Africa. A two-group randomized pilot study was conducted, comparing a women-focused intervention for methamphetamine use and related sexual risk behaviors to a psychoeducational condition. Participants were pregnant women who used methamphetamine regularly, had unprotected sex in the prior month, and were HIV-negative. Primary maternal outcomes were methamphetamine use in the past 30 days, frequency of unprotected sexual acts in the past 30 days, and number of antenatal obstetrical appointments attended. Primary neonatal outcomes were length of hospital stay, birth weight, and gestational age at delivery. Of the 57 women initially potentially eligible, only 4 declined to participate. Of the 36 women who were eligible and enrolled, 92% completed all four intervention sessions. Women in both conditions significantly reduced their methamphetamine use and number of unprotected sex acts. Therefore, delivering comprehensive interventions to address methamphetamine use and HIV risk behaviors among methamphetamine-using pregnant women is feasible in South Africa. Further testing of these interventions is needed to address methamphetamine use in this vulnerable population.

## 1. Introduction

Substance use during pregnancy is a critical health care concern. In Cape Town, South Africa, high rates of methamphetamine use have been found among women of childbearing age [1–3], including pregnant women [4]. Methamphetamine use is associated with several deleterious short- and long-term physical and psychological effects. A wide variety of physical effects have been associated with methamphetamine use, including respiratory and cardiac problems, palpitations, tremors, convulsions, stroke, and an increased risk of death [5]. Furthermore, its use is associated with risky sexual activities that place women at risk for acquiring HIV [1, 3, 6]. Psychological effects can be pronounced and include hallucinations, delusions, paranoia, and amphetamine psychosis [7]. Methamphetamine use has a high dependence risk and an extended withdrawal period, with frequent relapse. Prenatal stimulant exposure has been associated with being born small for gestational age [8], a risk factor for later developmental problems [9, 10] and poorer neurobehavioral outcomes [11].

Nonetheless, there are no substance use treatment models in South Africa that are tailored to address the unique needs of pregnant women who use methamphetamine. This study reports on the initial feasibility and acceptability of a women-focused intervention for methamphetamine use and related sexual risk behaviors among pregnant South African women. We blended two evidenced-based models to create an integrated drug treatment and HIV prevention model.

Reinforcement-Based Treatment (RBT), an efficacious comprehensive drug treatment model, was adapted to treat methamphetamine-using Coloured (mixed race ancestry) South African women. RBT's theoretical underpinnings include both operant conditioning principles (behavior is a reaction based on past consequences of actions [12]) and social learning theory (behavior is shaped by understanding of the relationship between the behavior and receiving a reward or punishment and the value placed on that reward or punishment [13]). Previous studies have shown that RBT reduced stimulant use in non pregnant patients relative to those patients randomly assigned to a control condition [14–17]. RBT, refined for stimulant-abusing pregnant patients enrolled in comprehensive care, also yielded positive results [17]: relative to comprehensive care alone, RBT had significantly greater rates of abstinence from stimulants. In addition, RBT has improved maternal drug treatment (e.g., reduced drug use and increased prenatal care utilization) and neonatal outcomes (e.g., length of hospitalization) in impoverished stimulant-using pregnant African American women [17, 18].

The Women's Health CoOp (WHC), a women-focused intervention found to be efficacious in Cape Town, South Africa [19], was integrated into RBT. The WHC intervention includes elements regarding gender roles, cultural context, knowledge about substance use and abuse, and skills for empowering women to reduce their sexual risk behavior and included role-playing and rehearsal to build self-efficacy and mastery for applying these skills. Female peer interventionists helped women develop a personalized action plan for applying new HIV sexual risk reduction skills in condom use and sexual negotiation and for reducing their drug use. Because this brief intervention has shown efficacy in South Africa, pairing it with RBT's efficacy in reducing drug use among pregnant women represented an ideal comprehensive approach to treating methamphetamine use and preventing sexual risk behaviors among pregnant, vulnerable women in South Africa.

The purpose of the present study was to report the results of a small-scale randomized pilot study with pregnant Coloured women (*N* = 36) designed to determine the initial feasibility and acceptability of our integrated RBT+WHC model relative to a psychoeducational control condition in terms of their respective impacts on primary *maternal outcomes* of methamphetamine use, unprotected sex, and (c) antenatal care and primary *neonatal outcomes* of (d) length of hospital stay, (e) birth weight, and (f) gestational age at delivery.

## 2. Methods

IRB approval was granted by the Institutional Review Boards of RTI International and Stellenbosch University's Faculty of Health Sciences.

### 2.1. Research and Community Setting

Participants were seen in a project field office located in Cape Town. This office had both private and communal rooms and was located in a community setting easily accessible to participants.

### 2.2. Participants

Recruitment took place in designated Cape Town township communities by outreach workers using targeted sampling methods, as well as by snowball sampling. Outreach workers screened women for initial study eligibility. To be eligible to participate in the study, participants had to be pregnant, 18 years of age or older, self-identify as Coloured (a cultural grouping of mixed race ancestry), live in predominantly Coloured township communities, self-report using methamphetamine in the past 30 days and report using methamphetamine regularly for at least the past six months, be willing to enter drug treatment, self-report unprotected sex in the past 30 days, be HIV-negative, and provide verifiable locator information for follow-up interview.

### 2.3. Procedures

Following recruitment, eligibility determination, written consent to participate, and collection of biological specimens to test for HIV, pregnancy, and alcohol and drug use, participants were administered a questionnaire by an interviewer. Following completion of this baseline assessment, the interviewer opened an envelope that contained information regarding treatment assignment for the participant. Treatment assignment was determined by a block randomization procedure for each successive pair of participants. Study staff were not involved in the randomization process and had no knowledge of the assignment of a participant until her envelope was opened.

The participant then began her intervention. Intervention sessions (described below) occurred once weekly for four weeks, with make-up sessions available for missed appointments. All participants had a total of eight weeks from their intake session to complete all four intervention sessions. There were seven days separating each intervention session so that there was time for behavior change to occur in between sessions. Participants had more flexibility for the postpartum visit due to the uncertainty of when the baby would be born. The participant had 30 days from her expected due date to complete the postpartum visit.

### 2.4. Intervention Conditions

Participants were randomized to one of two intervention conditions: Reinforcement-Based Treatment plus Women's Health CoOp (RBT+WHC) or Psycho-Educational (PE). Both conditions consisted of four weekly sessions, of two hours each, provided by a trained interventionist. The content of the first hour was the same in both conditions, although it was delivered differently: in RBT+WHC the material was delivered in an interactive format, with input and activities on the part of the participant; in the PE condition, the material was presented in a didactic format in which the participant was passive throughout, although she could ask questions at any time she wished. The content of the second hour differed markedly between the two intervention conditions.

### 2.5. RBT+WHC Condition

RBT+WHC represents modifications to RBT that included adapting four RBT intervention modules to be maximally relevant to a Coloured South African population and integrating into these modules information from WHC regarding alcohol and drug abuse risks and how certain sex behaviors increase HIV risk; the context of sexual risk for women in South Africa and condom use skills; gender roles; power in relationships and negotiation of safe sexual practices; and interpersonal violence prevention.

The resulting four modules addressed are (1) pregnancy and parenting; (2) drugs and alcohol; (3) HIV prevention, gender inequality, and violence prevention; and (4) planning your future. Pregnancy and parenting covered didactic topics such as pregnancy—month by month; the childbirth process and postpartum recovery; and parenting skills—how to appropriately calm a child and using effective communication. Drugs and alcohol focused on the long and short-term effects of drugs and alcohol; the effects of drugs and alcohol on women; the effects of drugs and alcohol on the baby; and having a balanced life and thinking about seeking formal substance abuse treatment. HIV prevention, gender inequality, and violence prevention focused on understanding HIV and the spread of HIV; learning how to use male and female condoms; understanding how gender inequality affects South African women; and how to effectively communicate and negotiate safe sex practices and dealing with potentially violent situations. Planning your future focused on understanding how skills, hobbies, and interests can help a job search; how to look for a job; and how to interview for a job.

There were six active RBT components utilized during the second hour of each RBT+WHC session: individual peer counseling; behavioral contracting; graphing; reinforcers; functional assessment; and life plan. The goal of individual peer counseling was for the interventionist to assist the participant in identifying some of the different factors that contribute to her substance use and how to overcome these factors. Behavioral contracting utilized written agreements between the participant and the interventionist regarding what concrete steps a participant might take towards meeting her goal(s). Graphing provided a visual guideline to monitor intervention goals and could involve line or bar graphs of behaviors that a given participant considered either problematic (e.g., methamphetamine use) or aspirational (e.g., job seeking). Peer interventionists provided reinforcers to acknowledge the participant's progress throughout the study and involved verbal recognition, stickers on graphs, and certificates for negative urine screening test results. The functional assessment had as its basis the intake interview undertaken at the beginning of the second hour of the first session. It determined problem areas associated with substance use and was the basis for dynamic goal-setting with the participant over the remaining weeks of treatment. Based on the problems identified, women in this condition were given referrals and linked to agencies providing additional services. The life plan comprised a series of goals, introduced weekly, and then reviewed over the following week(s), in which the peer interventionist helped the participants set and meet goals in antenatal care attendance; methamphetamine reduction or abstinence; protected sex; and postpartum care. These individualized second-hour sessions were interactive and dynamic, and goals changed weekly based on the review of the functional assessment. Their overarching aim was to empower participants to take control of their lives and actions.

### 2.6. PE Condition

Because “usual care” models treating methamphetamine-using pregnant women do not exist in South Africa, a “usual care” condition for the comparison of treatment efficacy was not an option; moreover, we felt that it was unethical to compare our intervention to the “no care” standard. Thus, we decided to develop a “usual care” model, using the four intervention modules included in the RBT intervention. These modules were complemented by case management services delivered during the second hour of each of the four sessions. Hence, the PE condition offered potentially active treatment components. However, the delivery of the intervention modules in the PE condition differed markedly from their delivery in the RBT+WHC condition as the focus of the PE modules was simply to impart information to the participant, provide case management focused on a needs assessment, and discuss the participant's needs to make changes in her life. A referral for services was only given if the participant requested a referral and was limited to providing the participant with a list of agencies appropriate to her needs.

### 2.7. Interventionists

Training included a three-day workshop on RBT principles and RBT-specific topics, together with training in the WHC modules. Training for the PE condition included a two-day workshop that included one-and-a-half days on implementation of the four intervention modules and a half-day devoted to group facilitation training of patient-led groups and how such groups are distinct from RBT+WHC. Caseloads were distinct and separate for each condition for the two interventionists and varied between 4 and 12 cases at any time. The two interventionists provided both RBT+WHC and PE services. To maintain clarity between intervention modalities and goals for each participant, weekly one-on-one supervision of each interventionist was conducted on-site by the project director, who was experienced in both RBT and WHC. Possible cross-condition contamination was minimized by completing fidelity checklists on each participant following implementation of the interventions. Interventionists needed to maintain a 90% adherence rating on the checklist to avoid intensive corrective feedback. Sessions were audiotaped, and one taped session per week per interventionist was reviewed by the project director and used for feedback. Weekly supervision was also conducted in both conditions.

### 2.8. Measures

Participants were administered a battery of measures at baseline and follow-up assessments. The primary outcomes were derived from the following two instruments.

*Revised Risk Behavior Assessment (RRBA)*. The RRBA has multiple domains including sections on demographics and social characteristics, health knowledge, alcohol and drug use, sexual practices, power and empowerment, conflict and victimization, physical and mental health, HIV testing, and need for services. The RRBA has shown excellent reliability in past research and has been used in multiple studies with substance-using women in South Africa [2, 6, 20].

*Mother and Infant Delivery Form (MIDF)*. The MIDF collected data from the medical chart of the participant and her neonate, including date and time of hospital admission, date and time and birth, physical birth parameters, and birth complications.

*Participant Satisfaction Form (PSF).* The PSF was an evaluation form completed by participants at the end of the intervention. It included questions that elicited information about what aspects of the intervention participants found acceptable and what aspects they might have found unacceptable.

### 2.9. Outcomes

There were six primary outcomes chosen for study examination: maternal outcomes of (1) methamphetamine use in the past 30 days (number of days); (2) frequency of unprotected sexual acts in the past 30 days (number of times); and (3) number of antenatal obstetrical appointments attended; and neonatal outcomes of (4) length of hospital stay (days), (5) birth weight (g), and (6) gestational age at delivery (weeks). The maternal outcomes were derived from the RRBA, while the neonatal outcomes came from the MIDF. Antenatal care was available to all participants through the midwife obstetric units (MOUs) located within primary health care clinics in the target communities. These MOUs offer free, publicly funded antenatal, delivery and postnatal services to all pregnant women who depend on public health services.

### 2.10. Statistical Analyses

Given that methamphetamine use in the past 30 days, frequency of unprotected sexual acts in the past 30 days, number of missed antenatal appointments, neonatal length of hospital stay, and estimated gestational age were count variables, they were assumed to follow a Poisson distribution, so an overdispersed Poisson regression was utilized for these outcomes. Birth weight was assumed to be normally distributed, and so an ordinary least squares regression analysis was utilized for this outcome. Separate analyses were conducted for each outcome measure. The between groups factor in all analyses was intervention condition (RBT+WHC *v*. PE). While the remaining outcomes were measured at posttreatment assessment only, methamphetamine use in the past 30 days and frequency of unprotected sexual acts in the past 30 days were measured both at baseline and posttreatment, so an additional within-subject factor of time (baseline *v*. posttreatment) was included in the model, and a generalized linear mixed model approach to their analyses was utilized. Finally, to assess acceptability of each intervention, we examined responses to the PSF questions.

## 3. Results

### 3.1. Feasibility of Participant Enrollment

The total number of potential participants screened was 293. Of these 293, 216 were ineligible because they reported no lifetime use of methamphetamine, 57 were ineligible as they reported no instances of unprotected sex with a male partner in the past 30 days, 15 were ineligible because they were younger than 18 years of age, 36 were ineligible because they were of another ethnic group, 9 were unwilling to be tested for pregnancy as part of their enrollment in the study, and 15 were ineligible because they self-reported being HIV-positive. Of the 57 potentially eligible participants, 21 were later found to be ineligible because they were unwilling to provide tracking information, were younger than 18 years of age, or were not pregnant, and 4 declined participation in the study because of a lack of interest. Some potential participants were ineligible for multiple reasons.

### 3.2. Participant Characteristics

Demographic and background information on the remaining 36 eligible participants is reported in Table 1. The conditions did not differ on any of these characteristics (all *P*s > .1). The sample as a whole is relatively young (between 18 and 35 years of age, inclusive, with only 6 women 30 years of age or older), with 22 having an education through grades 9–12. Of the 35 participants with a main sex partner, 16 were married or living with him. The duration of these main partnerships ranged from five months to 22 years, and all 35 women indicated that they believed that this partner likewise considered them to be their main sex partner. Age range for first vaginal intercourse was between 13 and 21 years of age. Age at first methamphetamine use ranged between 14 and 30 years of age, with a maximum of 132 months of use. The number of attempts to quit methamphetamine use ranged from 1 to 20, with eight participants indicating no lifetime attempts to quit methamphetamine use.

### 3.3. Acceptability of the Intervention

Overall, 33 of the 36 participants completed all four intervention sessions, with one participant missing three sessions, one participant missing one session, and another missing one session to give birth. The majority of participants in both the RBT+WHC and PE conditions experienced the interventions as helpful and did not have suggestions about how to improve the interventions. In the PE condition, five participants thought that the intervention program was too short and two wished they could continue with the program. In addition, two people wanted more pictures and illustrations, and two people did not like the pictures of the STIs that were included in the interventions. Twelve participants in the RBT+WHC condition reported that they “learned a lot” and “liked everything” about the intervention. They particularly liked the help they received with condom use, communication skills, and job seeking. They expressed a desire to extend the benefits of the program to their social networks and partners. However, two participants thought there were not enough sessions, one participant thought that the sessions were too long in duration and should be shorter, one did not like the STI pictures, and another did not like the module on violence prevention.

### 3.4. Outcomes

*Maternal Outcomes.* Neither the intervention condition × time interaction effect nor the intervention condition main effect were significant for either methamphetamine use in the past 30 days or frequency of unprotected sexual acts in the past 30 days (all *P*s > .2). However, the time effect was significant for both variables (see Table 2). Methamphetamine use in the past 30 days decreased from baseline to posttreatment (*M* = 10.8 (SE = 1.7) at baseline; *M* = 4.8 (SE = 1.2) at posttreatment) and frequency of unprotected sexual acts in the past 30 days also decreased (*M* = 4.4 (SE = 0.7) at baseline; *M* = 1.4 (SE = 0.4) at posttreatment), with a corresponding decrease in the percentage of unprotected sexual acts from 90.1% at baseline to 74.9% at posttreatment. The conditions did not differ on the number of antenatal appointments attended (*P* > .2). 

*Neonatal Outcomes.* There were no differences between the intervention conditions on any of the three neonatal outcomes (all *P*s > .1; see Table 2).

## 4. Discussion

Results from this initial feasibility study indicate that methamphetamine-using pregnant women in South Africa are willing to enroll in and complete an intervention aimed at reducing their methamphetamine use. The opportunity to participate in a research study that provided treatment for their methamphetamine use was highly appealing to the participants who were recruited, witnessed by the fact that only 4 of the 57 potentially eligible participants (7%) indicated that they were not interested in participation. This high rate of enrollment on the part of eligible participants, together with the fact that 92% of the women completed all four intervention sessions, indicate that both interventions have a high degree of acceptability for methamphetamine-using pregnant women. These enrollment and completion rates are in stark contrast to the fact that only 14% of the 36 participants who enrolled in the study indicated that they had *ever* been in substance use treatment. Moreover, participants in both conditions provided generally positive feedback on the interventions, reinforcing their acceptability. However, we also received feedback that there were parts of the interventions that challenged them and made them feel uncomfortable. Future studies may need to examine the role and importance of these intervention components.

The fact that a relatively large number of potential participants were screened out of participation in the trial should not be taken to mean that an intervention such as RBT+WHC is not needed. The trial screening methodology and eligibility criteria necessitated a very well-defined sample of pregnant women who used methamphetamine. Moreover, the sample was restricted to Coloured women. Recent research in Cape Town suggests that the use of methamphetamine among women, particularly pregnant women, presents a public health concern [4, 19, 21].

It is also the case that participants changed from baseline to posttreatment on two of the three maternal outcome measures, reducing both their past 30-day methamphetamine use and frequency of unprotected sex. Although such changes cannot be unambiguously attributed to a treatment effect, findings do suggest that the participants as a group were motivated to change their risky behavior and that the content of the interventions appears to be of some benefit to participants. Such an interpretation, if substantiated, would provide additional support for the further development of interventions for methamphetamine use and prevention of HIV among pregnant women in South Africa.

Conversely, failure to find significant differences between the intervention conditions does not suggest that efforts to further develop either or both interventions should be abandoned, as the trial was not powered to detect such differences. Although they fail to reach significance, results for length of hospital stay and birth weight favor the RBT+WHC condition over the PE condition. Future research that employs a comprehensive women-centered intervention such as RBT+WHC in a larger sample may wish to focus on these variables as primary outcomes of interest.

Consistent with previous studies [4], antenatal care utilization remained low in both conditions, despite the fact that antenatal care use was emphasized in both conditions. Six participants did not use antenatal care, and the maximum number of antenatal visits was 9, by one participant. Such low rates of antenatal care utilization are not surprising, as the general population of pregnant women in Cape Town often delay seeking antenatal care until late in their pregnancy and/or underutilize these services unless they have specific complications [22, 23]. Nonetheless, in the Western Cape, more than 90% of pregnant women do attend an antenatal clinic, and for this study, there are 2 Midwife Obstetric Units and two Basic Antenatal Care clinics available in their community within a radius of 7 km from the study site. Thus, this low rate of utilization is a concern because failure to use antenatal care is associated with poor birth outcomes, especially among drug-using women who are more at risk for pregnancy-related complications and whose infants are at greater risk for infant morbidity and mortality [9, 10]. One plausible explanation for these low rates of antenatal care utilization may lie in findings from previous South African research that have described stigmatizing and pejorative attitudes of midwives towards pregnant women suspected of substance use [23, 24]. In fact, we suspect that some of our difficulties in finding eligible women who used methamphetamine were because women were afraid to self-disclose their methamphetamine use due to fears of being stigmatized. Regardless of the reason, our findings suggest that more effort is needed to link pregnant women who use methamphetamine with antenatal care.

There are four important limitations to the present study. The first limitation is the small sample size. The study planned to use a small sample because its primary focus was on feasibility and acceptability. Thus, any conclusion regarding the failure to find differences in support of either intervention is premature because the study was not powered to determine the differential efficacy of the two interventions. The second limitation is the characteristics of the sample recruited for participation. Although all women clearly had a history of methamphetamine use, some for extended periods, none of the women used methamphetamines at a high rate at study entry. Moreover, the women appeared to be in long-term, stable relationships with their main sex partner, and so the rate of condom use at study entry was quite low. The third limitation is that the study did not include a no-intervention control condition. Thus, we are unable to discount the possibility that participants may have improved simply due to attentional factors unrelated to treatment, their increased motivation as a result of enrolling in the study, and/or expectancy effects. Our findings suggest that inclusion of a no-treatment control condition should be a priority in future research. The fourth limitation is that maternal methamphetamine use was not verified by urine screening at the point of initial screening. This is a potentially important limitation because women who chose not to disclose their methamphetamine use due to the stigma attached to methamphetamine use in this population would not have been recruited into the study.

Findings from the present study suggest that an intervention aimed at reducing methamphetamine use is feasible to implement and acceptable to participants. Consequently, further development of such interventions among pregnant South Africans is warranted. Low rates of antenatal care utilization would suggest that infants birthed by this population are at increased risk of morbidity and mortality. In addition, low rates of condom use suggest that these women are at heightened risk for sexually transmitted diseases, including HIV. The development of comprehensive, women-focused interventions for methamphetamine-using pregnant women in this region is a priority. Vulnerable pregnant women in South Africa are guardians of the next generation and deserve the full attention of applied researchers, policy makers, and service providers.

## Figures and Tables

**Table 1:** Demographic and selected background characteristics (*N* = 36).

Variable	Total sample (*N* = 36)		RBT+WHC condition (*n* = 17)		PE condition (*n* = 19)	
	*n* (%)	M (SD)	*n* (%)	M (SD)	*n* (%)	M (SD)
Age (in years)		24.6 (4.6)		24.2 (4.9)		24.9 (4.3)
Highest grade completed at school		9.1 (1.5)		9.2 (1.7)		9.1 (1.3)
Relationship status						
Married to main sex partner	3 (8%)		1 (6%)		2 (11%)	
Living with a main sex partner	13 (36%)		8 (47%)		5 (26%)	
Having a main sex partner but not living with him	19 (53%)		8 (47%)		11 (58%)	
No main sex partner	1 (3%)				1 (5%)	
Number of months with main sex partner		65.5 (54.9)		73.5 (60.8)		58.0 (49.4)
Age at first vaginal intercourse		16.6 (2.2)		16.5 (2.0)		16.7 (2.4)
Ever in your lifetime been in alcohol/drug rehabilitation: yes	5 (14%)		2 (11%)		3 (16%)	
Age at first methamphetamine use		19.3 (4.0)		19.6 (4.5)		19.1 (3.5)
Number of lifetime months of methamphetamine use^†^		53.7 (31.7)		47.6 (29.6)		60.3 (33.6)
How many times tried to stop using methamphetamine		3.2 (4.5)		3 (4.1)		3.5 (4.8)
Methamphetamine use after becoming pregnant						
More often	7 (19%)		3 (18%)		4 (21%)	
About the same	5 (14%)		2 (12%)		3 (16%)	
Less often	23 (64%)		12 (71%)		11 (58%)	
Quit	1 (3%)		0 (0%)		1 (5%)	
Considered quitting methamphetamine after becoming pregnant: yes	31 (86%)		13 (76%)		18 (95%)	
How many previous pregnancies		1.9 (1.3)		1.9 (1.4)		1.9 (1.2)
Was this pregnancy planned: yes	10 (28%)		6 (35%)		4 (21%)	
How many weeks along before finding out about pregnancy^‡^		11.9 (7.1)		13.1 (7.1)		10.8 (7.0)
Had a gynecological exam for this pregnancy: yes	22 (61%)		9 (53%)		13 (68%)	

^†^5 participants declined to answer this question. ^‡^4 participants were unable to remember with any accuracy and so failed to answer.

**Table 2:** Test statistics, *P* values, estimated marginal means, and standard errors for primary outcomes (*N* = 36).

Outcome	Effect of interest	Test statistic	*P*	Means (standard errors)	
				RBT+WHC condition (*n* = 17)	PE condition (*n* = 19)
Maternal outcomes					
Past 30 day number of times of methamphetamine use	Treatment × time	*χ* ^2^(1) = 0.1	.81	Baseline: 9.2 (2.2)	Baseline: 12.6 (2.5)
				Posttreatment: 3.8 (1.6)	Posttreatment: 5.9 (1.7)
Past 30 day number of times unprotected vaginal intercourse	Treatment × time	*χ* ^2^(1) = 0.5	.49	Baseline: 4.6 (1.1)	Baseline: 4.2 (1.0)
				Posttreatment: 1.1 (0.6)	Posttreatment: 1.6 (0.6)
Number of antenatal obstetrical care visits attended	Treatment	*χ* ^2^(1) = 1.5	.22	1.6 (0.3)	2.5 (0.2)
Neonatal outcomes					
Neonatal length of hospital stay^†,‡^	Treatment	*χ* ^2^(1) = 2.5	.11	2.1 (0.9)	4.2 (0.4)
Birth weight^†^	Treatment	*F*(1, 31) = 0.6	.46	2900.7 (124.1)	2774.3 (113.3)
Gestational age at delivery^†^	Treatment	*χ* ^2^(1) = 0.1	.75	37.5 (0.02)	37.2 (0.02)

^†^1 participant completed only the first session and then dropped out of the study, 1 participant miscarried, and 1 participant had her infant die shortly after birth and denied permission for access to her infant's record ^‡^and, in addition, the infant discharge date was not available in medical records for 2 other infants.
